# Migraine after stellate ganglion block: A case report

**DOI:** 10.1002/ccr3.8119

**Published:** 2023-10-31

**Authors:** Xiaowei Chi, Bin Jia, Qiang Fu

**Affiliations:** ^1^ Department of Anesthesiology, Third People's Hospital of Chengdu Southwest Jiaotong University Chengdu China; ^2^ Department of Cardiac Surgery, Third People's Hospital of Chengdu Southwest Jiaotong University Chengdu China

**Keywords:** anesthesia, case report, migraine headache, stellate ganglion block

## Abstract

Stellate ganglion block‐induced ipsilateral migraines are rare. We present a typical case detailing its developmental process. Abnormalities in the autonomic nervous system control and vascular and neural mechanisms may play crucial roles in the manifestation of these migraines. Postprocedural migraines necessitate anesthesiologists' awareness during stellate ganglion blocks.

## Graphical abstract

Ultrasonic exploration of stellate ganglion.

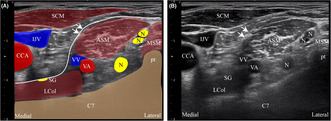

## 1 INTRODUCTION

The stellate ganglion is formed by the fusion of the inferior cervical and first thoracic sympathetic ganglion. It lies anterior to the transverse process of C7 and superior border of the first rib and posteromedial to the carotid artery. The ganglion is covered by prevertebral fascia, which is a layer of deep cervical fascia. Sympathetic fibers for the head, neck, thoracic viscera, and upper limbs synapse in the stellate ganglion. Therefore, the ganglion is a target location to block for the treatment of pain and other conditions affecting these anatomical areas.^1^ A stellate ganglion block (SGB) can be performed safely using anatomical landmarks, or under fluoroscopy or ultrasound guidance. The SGB target area is usually the middle cervical sympathetic ganglion on the surface of the longus colli at the level of C6, with the injectate flowing caudally.

Currently, ultrasound‐guided SGB, performed to safely block the sympathetic ganglia innervating the head, neck, face, and upper limbs, functions through vasodilation of the target area. It can also be used for systemic diseases such as insomnia and autonomic imbalance.^2^, ^3^ Previous studies confirmed that cerebrovascular resistance can be sustainably reduced, cerebral blood flow can be moderately increased, and cerebrovascular spasms can improve after cervical sympathectomy or sympathetic ganglion block.^1^ These findings are consistent with those of animal studies.^4^, ^5^ Other studies have shown that reactions, such as decreased cerebral vascular tone, are present in patients with abnormal intracranial physiological conditions, such as cerebral vasospasm,^1^, ^6^ and in those without such conditions.^7^

Our literature search revealed only two other patients with migraine episodes similar to those in the current case.^8^, ^9^ However, unlike our patient, the two patients experienced migraines accompanied by an aura, and the migraines persisted for several months. Therefore, this report presents our case of a patient who experienced migraines without auras after undergoing SGB for insomnia.

## 2 CASE REPORT

A mid‐40s man was admitted with complaints of difficulty falling asleep and insomnia. His Pittsburgh Sleep Quality Index (PSQI)^8^ score was 12, signifying poor sleep quality, and his Insomnia Severity Index (ISI)^9^ score was 19, indicating clinical insomnia. Past medical and family histories were normal. Despite taking the sedative zolpidem for over 5 years, he found traditional medication for insomnia ineffective. He urgently needed to stop taking the medication and desired non‐pharmacological treatment to address the sleep difficulties. After obtaining procedural informed consent from the patient, we scheduled an ultrasound‐guided SGB. The general procedure for SGB is discussed in the following section.

### 2.1 Procedure for SGB

**FIGURE 1:**
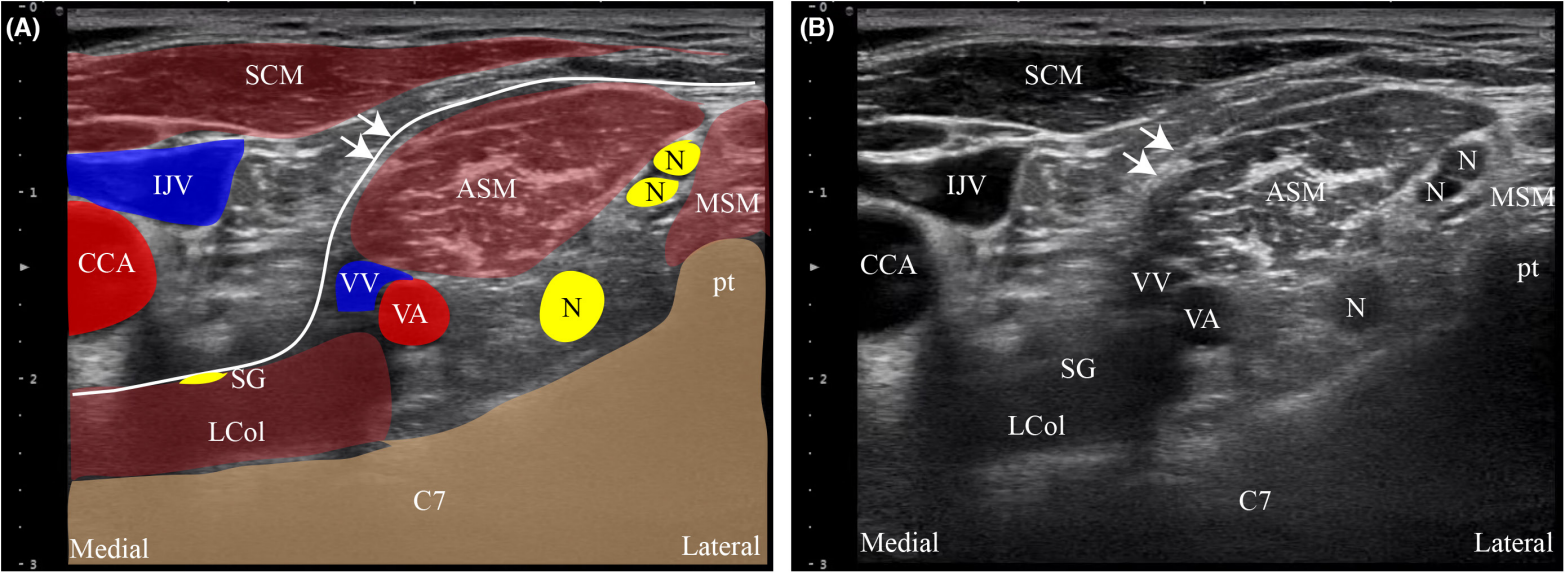
Short axis sonogram at C7. (A, B) The transducer is moved slowly laterally and cephalad until the characteristic posterior tubercle of C7 appears in the image. The solid arrow shows the anterior vertebral fascia. The stellate ganglion is located on the deep surface of the anterior fascia and the surface of the longus colli muscle. ASM, anterior scalene muscle; CCA, common carotid artery; IJV, internal jugular vein; Lcol, longus colli muscle; MSM, middle scalene muscle; N, nerve; pt, posterior tubercle; SCM, sternocleidomastoid muscle; SG, stellate ganglion; VA, vertebral artery; VV, vertebral vein.

The patient is positioned supine in the anesthesia outpatient treatment room with the head turned to the left and the neck fully exposed. After standard disinfection, a high‐frequency linear array probe for color Doppler ultrasound identifies the right C7 vertebrate's transverse process at the cricoid cartilage plane, and moved slightly toward the midline. Various anatomical structures like the carotid artery, internal jugular vein, thyroid gland, trachea, esophagus, and longus colli/cervicis are pinpointed. The stellate ganglion is located on the longus colli/cervicis surface (Figure 1). Under ultrasound guidance, the SGB is performed using an in‐plane injection method. The injection needle's tip halts after passing through the prevertebral fascia and reaching the longus colli/cervicis surface, followed by injecting 4 mL of 0.25% ropivacaine. Successful block is confirmed by the presence of Horner's syndrome signs including ipsilateral myosis, ptosis, enophthalmos, nasal congestion, and facial anhidrosis.

### 2.2 Post procedure

**FIGURE 2:**
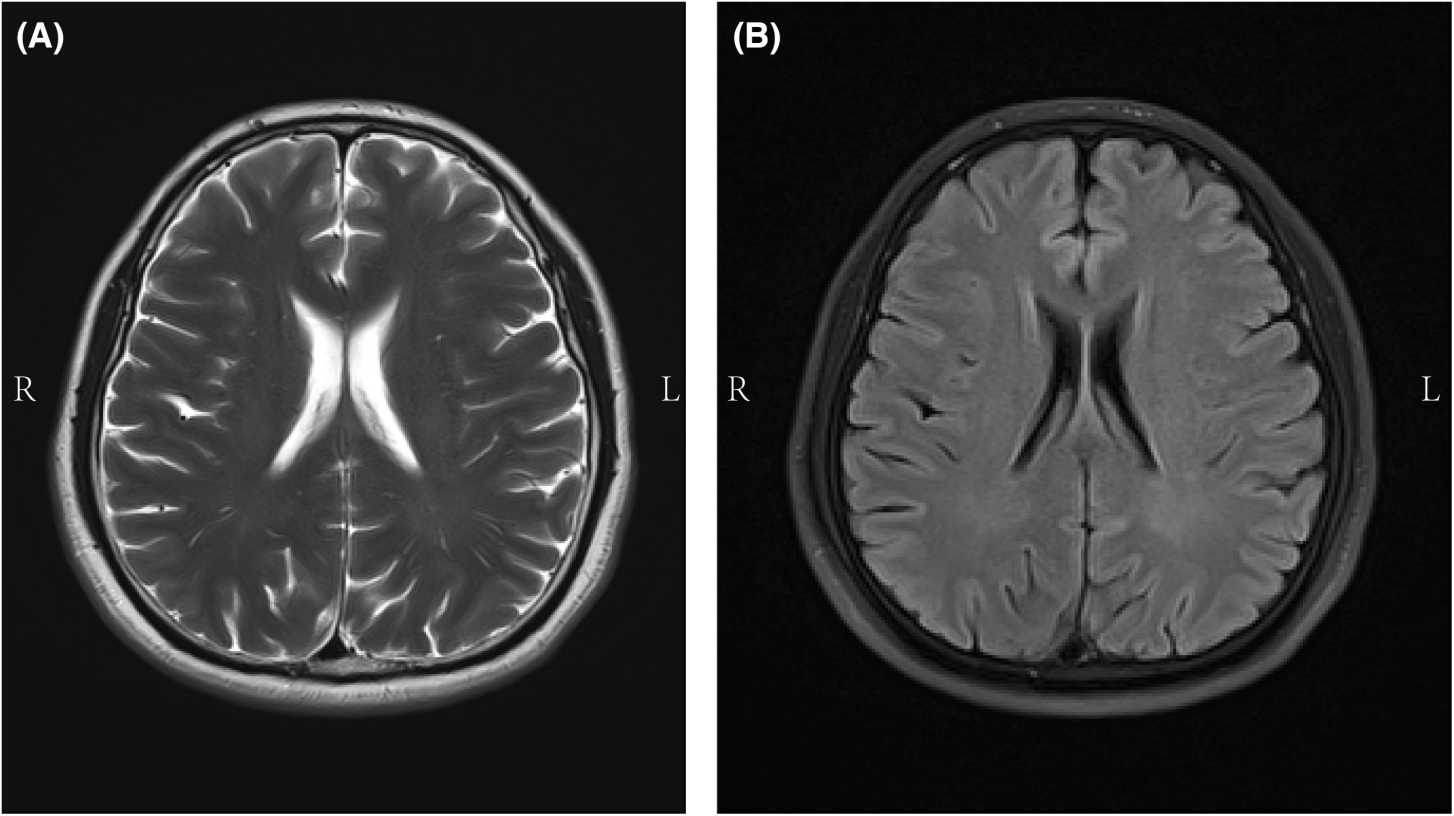
Brain MRI. Axial section of the lateral ventricle shows no obvious abnormalities. (A) T2‐weighted image (T2WI); (B) T1‐weighted image (T1WI). L, left; MRI, magnetic resonance imaging; R, right.

Thirty minutes following SGB, our patient experienced a severe pulsatile headache in the right temporal lobe area (temple) accompanied by nausea. Vital signs were as follows: HR, 93 bpm; BP, 126/69 mm Hg; and SpO_2_, 99%. Although the pain was slightly reduced after oral administration of a nonsteroidal anti‐inflammatory drug (ibuprofen), the headache persisted for over 20 h. The patient underwent head magnetic resonance imaging (MRI) the next day, which was unremarkable, showing no organic lesions in the intracranial area (Figure 2). On the following day, a neurologist conducted a consultation with the patient. During the subsequent 2 weeks, the patient experienced 2–3 similar migraine episodes per day and was otherwise feeling well between these episodes. Physical signs and symptoms were consistent with *migraine without aura* (International Headache Society [IHS] definition^10^) as defined by a neurologist. Approximately 2½ weeks later (4½ weeks from migraine onset), the patient returned for a follow‐up appointment. As of that appointment, the patient has had no further migraines.

## 3 DISCUSSION

Migraine is a complex neurological disease characterized by recurrent episodes of moderate‐to‐severe pain intensity headaches.^11^ Despite its prevalence, the underlying pathophysiological mechanisms remain elusive.^11^, ^12^

The current patient had no history of migraines or other chronic headaches. The signs and symptoms of the ipsilateral migraine without aura developed after ultrasound‐guided SGB for insomnia. Our literature search revealed two other cases in which the patients experienced migraines after SGB.^13^, ^14^ Those patients had auras accompanying the migraines, and the migraines persisted for several months. The differences between those patients and the current patient are shown in Table 1. There are differences in volume of local anesthetics between these patients. The drugs and concentrations used were different which suggest this phenomenon might be dose and volume independent.

**TABLE 1:** Cases of migraine attacks after SGB.

	First patient^11^	Second patient^12^	Current patient
Report year	1996	2006	2023
Sex	Male	Female	Male
Age (years)	56	41	Mid‐40s
Diagnosis	Left hand reflex sympathetic dystrophy	Left hand complex regional pain syndrome	Insomnia
SGB	Left side, 0.25% bupivacaine, 12 mL	Left side, 0.375% ropivacaine, 8 mL	Right side, 0.25% ropivacaine, 4 mL
Horner's syndrome	Yes	Yes	Yes
History of migraine headache or any other headache condition	History of meningitis 10 prior, when global headaches lasted for 10 days	None	None
Migraine aura	Blurry vision	Blurry vision and speech disturbance	None
Headache area	Bifrontal	Left frontal	Right temporal lobe area (temple)
Headache severity	Severe, excruciating pain intensity	Severe pain intensity	Moderate‐to‐severe pain intensity
Accompanied symptoms	Nausea, photophobia, and phonophobia	Nausea and phonophobia	Nausea
Headache frequency	Phase 1: 2–3 times per week, lasting for 4 weeks; Phase 2: Once every 8 weeks, lasting for 20 weeks	Once per 2 weeks, lasting for 12 weeks	2–3 times per day, lasting for 2 weeks
Medical treatment	Acetaminophen, sumatriptan succinate, butorphanol nasal spray	Sumatriptan succinate	Ibuprofen
Follow‐up/Prognosis	Resolved completely	Resolved completely	Resolved completely

Abbreviation: SGB, stellate ganglion block.

Our patient experienced an ipsilateral migraine after undergoing a right stellate nerve block. Upon initial onset, we considered that the migraine may have resulted from the sympathetic block causing vasodilation of the ipsilateral head vasculature. However, after the disappearance of the acute sympathetic inhibition, the migraines persisted intermittently and lasted for 2 weeks before disappearing. The two previously reported patients experienced post sympathetic block migraines which lasted intermittently for several months (Table 1) before disappearing. We speculate that the mechanical expansion of vascular smooth muscle cells may activate nociceptors around blood vessels and release pain‐promoting and proinflammatory substances. Thus, after complete recovery from vasodilation, intermittent migraines can persist.

## 4 CONCLUSION

In conclusion, ipsilateral migraines induced by SGB are rare. Therefore, the exact mechanisms underlying their occurrence remain unclear. We suggest that abnormalities in autonomic nervous system control and vascular and neural mechanisms contribute to migraine occurrence. More reported cases are required to further elucidate the mechanisms of SGB‐induced migraines.

## AUTHOR CONTRIBUTIONS

**Xiaowei Chi:** Conceptualization; methodology; writing – original draft. **Bin Jia:** Conceptualization; methodology; writing – original draft. **Qiang Fu:** Writing – review and editing.

## FUNDING INFORMATION

This study was funded by Medical Research Project of Chengdu Medical Association (2021385).

## CONFLICT OF INTEREST STATEMENT

The authors declare that the research was conducted in the absence of any commercial or financial relationships that could be construed as a potential conflict of interest.

## ETHICS STATEMENT

This study was approved by the Medical Ethics Committee of The Third People's Hospital of Chengdu and was conducted in accordance with the Declaration of Helsinki.

## CONSENT

Written informed consent was obtained from the patient to publish this report in accordance with the journal's patient consent policy.

## Data Availability

The data that support the findings of this study are available from the corresponding author upon reasonable request.

## References

[1] ter Laan M , van Dijk JMC , Elting JWJ , Staal MJ , Absalom AR . Sympathetic regulation of cerebral blood flow in humans: a review. Br J Anaesth. 2013;111:361‐367. doi:10.1093/bja/aet122 23616589

[2] Haest K , Kumar A , Van Calster B , et al. Stellate ganglion block for the management of hot flashes and sleep disturbances in breast cancer survivors: an uncontrolled experimental study with 24 weeks of follow‐up. Ann Oncol. 2012;23(6):1449‐1454. doi:10.1093/annonc/mdr478 22039079

[3] Uchida K , Tateda T , Hino H . Novel mechanism of action hypothesized for stellate ganglion block related to melatonin. Med Hypotheses. 2002;59(4):446‐449. doi:10.1016/s0306-9877(02)00158-5 12208186

[4] Hu N , Wu Y , Chen BZ , Han JF , Zhou MT . Protective effect of stellate ganglion block on delayed cerebral vasospasm in an experimental rat model of subarachnoid hemorrhage. Brain Res. 2014;1585:63‐71. doi:10.1016/j.brainres.2014.08.012 25128600

[5] Dagistan Y , Kilinc E , Balci CN . Cervical sympathectomy modulates the neurogenic inflammatory neuropeptides following experimental subarachnoid hemorrhage in rats. Brain Res. 2019;1722:146366. doi:10.1016/j.brainres.2019.146366 31401069

[6] Wendel C , Scheibe R , Wagner S , et al. Decrease of blood flow velocity in the middle cerebral artery after stellate ganglion block following aneurysmal subarachnoid hemorrhage: a potential vasospasm treatment? J Neurosurg. 2019;133:773‐779. doi:10.3171/2019.5.JNS182890 31398704

[7] Gupta MM , Bithal PK , Dash HH , Chaturvedi A , Mahajan RP . Effects of stellate ganglion block on cerebral haemodynamics as assessed by transcranial Doppler ultrasonography. Br J Anaesth. 2005;95:669‐673. doi:10.1093/bja/aei23 16155036

[8] Buysse DJ , Reynolds CF , Monk TH , Berman SR , Kupfer DJ . The Pittsburgh sleep quality index: a new instrument for psychiatric practice and research. Psychiatry Res. 1989;28:193‐213. doi:10.1016/0165-1781(89)90047-4 2748771

[9] Bastien CH , Vallières A , Morin CM . Validation of the insomnia severity index as an outcome measure for insomnia research. Sleep Med. 2001;2:297‐307. doi:10.1016/S1389-9457(00)00065-4 11438246

[10] Headache classification Committee of the International Headache Society (IHS) the international classification of headache disorders, 3rd edition. Cephalalgia. 2018;38:1‐211. doi:10.1177/0333102417738202 29368949

[11] Do TP , Hougaard A , Dussor G , Brennan KC , Amin FM . Migraine attacks are of peripheral origin: the debate goes on. J Headache Pain. 2023;24:3. doi:10.1186/s10194-022-01538-1 36627561 PMC9830833

[12] Levy D , Moskowitz MA . Meningeal mechanisms and the migraine connection. Annu Rev Neurosci. 2023;46:39‐58. doi:10.1146/annurev-neuro-080422-105509 36913712 PMC11412714

[13] Lehmann LJ , Warfield CA , Bajwa ZH . Migraine headache following stellate ganglion block for reflex sympathetic dystrophy. Headache. 1996;36:335‐337. doi:10.1046/j.1526-4610.1996.3605335.x 8682679

[14] Beleña J , Petersen I , Cabeza R , Núñez M , Vidal A . Migraine headache: a rare complication after cervicothoracic block. J Headache Pain. 2006;7:367‐368. doi:10.1007/s10194-006-0330-2 17058040 PMC3468177

